# Convergence of the Transcriptional Responses to Heat Shock and Singlet Oxygen Stresses

**DOI:** 10.1371/journal.pgen.1002929

**Published:** 2012-09-13

**Authors:** Yann S. Dufour, Saheed Imam, Byoung-Mo Koo, Heather A. Green, Timothy J. Donohue

**Affiliations:** 1 Department of Bacteriology, University of Wisconsin–Madison, Madison, Wisconsin, United States of America; 2 BACTER Institute, University of Wisconsin–Madison, Madison, Wisconsin, United States of America; 3 Program in Cellular and Molecular Biology, University of Wisconsin–Madison, Madison, Wisconsin, United States of America; 4 Department of Microbiology and Immunology, University of California San Francisco, San Francisco, California, United States of America; Agency for Science, Technology, and Research, Singapore

## Abstract

Cells often mount transcriptional responses and activate specific sets of genes in response to stress-inducing signals such as heat or reactive oxygen species. Transcription factors in the RpoH family of bacterial alternative σ factors usually control gene expression during a heat shock response. Interestingly, several α-proteobacteria possess two or more paralogs of RpoH, suggesting some functional distinction. We investigated the target promoters of *Rhodobacter sphaeroides* RpoH_I_ and RpoH_II_ using genome-scale data derived from gene expression profiling and the direct interactions of each protein with DNA *in vivo*. We found that the RpoH_I_ and RpoH_II_ regulons have both distinct and overlapping gene sets. We predicted DNA sequence elements that dictate promoter recognition specificity by each RpoH paralog. We found that several bases in the highly conserved TTG in the −35 element are important for activity with both RpoH homologs; that the T-9 position, which is over-represented in the RpoH_I_ promoter sequence logo, is critical for RpoH_I_–dependent transcription; and that several bases in the predicted −10 element were important for activity with either RpoH_II_ or both RpoH homologs. Genes that are transcribed by both RpoH_I_ and RpoH_II_ are predicted to encode for functions involved in general cell maintenance. The functions specific to the RpoH_I_ regulon are associated with a classic heat shock response, while those specific to RpoH_II_ are associated with the response to the reactive oxygen species, singlet oxygen. We propose that a gene duplication event followed by changes in promoter recognition by RpoH_I_ and RpoH_II_ allowed convergence of the transcriptional responses to heat and singlet oxygen stress in *R. sphaeroides* and possibly other bacteria.

## Author Summary

An important property of living systems is their ability to survive under conditions of stress such as increased temperature or the presence of reactive oxygen species. Central to the function of these stress responses are transcription factors that activate specific sets of genes needed for this response. Despite the central role of stress responses across all forms of life, the processes driving their organization and evolution across organisms are poorly understood. This paper uses genomic, computational, and mutational analyses to dissect stress responses controlled by two proteins that are each members of the RpoH family of alternative σ factors. RpoH family members usually control gene expression during a heat shock response. However, the photosynthetic bacterium *Rhodobacter sphaeroides* and several other α-proteobacteria possess two or more paralogs of RpoH, suggesting some functional distinction. Our findings predict that a gene duplication event followed by changes in DNA recognition by RpoH_I_ and RpoH_II_ allowed convergence of the transcriptional responses to heat and singlet oxygen stress in *R. sphaeroides* and possibly other bacteria. Our approach and findings should interest those studying the evolution of transcription factors or the signal transduction pathways that control stress responses.

## Introduction

Transcriptional responses to stress are critical to cell growth and survival. In bacteria, stress responses are often controlled by alternative σ factors that direct RNA polymerase to transcribe promoters different from those recognized by the primary σ factor [1], [2]. Therefore, identifying the target genes for a particular alternative σ factor can help identify the functions necessary to respond to a given stress. For example, the transcriptional response to heat shock in *Escherichia coli* uses the alternative σ factor σ^32^ to increase synthesis of gene products involved in protein homeostasis or membrane integrity [3]. From available genome sequences, proteins related to *E. coli* σ^32^ are conserved across virtually all proteobacteria. This so-called RpoH family of alternative σ factors is characterized by a conserved amino acid sequence (the “RpoH box”) that is involved in RNA polymerase interactions [4], [5]. RpoH family members also possess conserved amino acid sequences in σ factor regions 2.4 and 4.2 that interact with promoter sequences situated approximately −10 and −35 base pairs upstream of the transcriptional start sites, respectively [6]. However, the definition of functional promoters for this family of alternative σ factor using only the presence or the extent of sequence identity for the predicted −10 and −35 binding regions is not a sufficient predictor of transcription activity [7].

While bacteria often possess many alternative σ factors, they usually possess only one member of the RpoH family. However, several α-proteobacteria, including *Brucella melitensis* [8], *Sinorhizobium meliloti* [9], [10], *Bradyrhizobium japonicum* [11], [12], *Rhizobium elti* [13] and *Rhodobacter sphaeroides* [14], possess two or more RpoH homologs. In some cases, one or more of these RpoH homologs completely or partially complement the phenotypes of *E. coli* Δ*rpoH* mutants, suggesting that these proteins can functionally interact with RNA polymerase and recognize similar promoter elements [8]–[11], [14], [15]. However, in the nitrogen-fixing plant symbiont *Rhizobium elti*, the Δ*rpoH_1_* mutant was sensitive to heat and oxidative stress while the Δ*rpoH_2_* mutant was sensitive to osmotic stress [13]. Therefore, the additional members of the RpoH family in α-proteobacteria may have roles in other stress responses.

Previous work demonstrated that either *R. sphaeroides* RpoH_I_ or RpoH_II_ can complement the temperature sensitive phenotype of an *E. coli* Δ*rpoH* mutant; that singly mutant *R. sphaeroides* strains lacking either *rpoH_I_* or *rpoH_II_* are able to mount a heat shock response; and that RNA polymerase containing either RpoH_I_ or RpoH_II_ can initiate transcription from a common set of promoters *in vitro* [14]–[16]. Combined, these observations suggest that RpoH_I_ and RpoH_II_ have some overlapping functions in *R. sphaeroides*. On the other hand, *in vitro* transcription assays identified promoters that were selectively transcribed by either RpoH_I_ or RpoH_II_ [14], [15]. Moreover, *rpoH_II_* is under direct transcriptional control of RpoE, a Group IV alternative σ factor that acts as the master regulator of the response of *R. sphaeroides* to singlet oxygen stress [17]–[19]. These later results and the recent observation that a Δ*rpoH_II_* mutant is more sensitive to singlet oxygen stress than the wild-type strain [15], [17] suggest that RpoH_I_ and RpoH_II_ also have distinct functions in *R. sphaeroides*. Finally, global protein profiles of *R. sphaeroides* mutants lacking *rpoH_I_*, *rpoH_II_*, or both genes, suggested that RpoH_I_ and RpoH_II_ have distinct and overlapping regulons [15], [17], [20]. However, the extent of genes that are direct targets for RpoH_I_ and RpoH_II_ is still unknown because past studies have been unable to distinguish direct from indirect effects on gene expression or identify all the direct targets for either of these σ factors.

In this study, we characterized the RpoH_I_ and RpoH_II_ regulons using a combination of expression microarrays, chromatin immunoprecipitation and computational methods which have been previously been shown to predict correctly direct targets for other alternative σ factors or DNA binding proteins [19], [21]. We found that the genes predicted to be common to the RpoH_I_ and RpoH_II_ regulons function in protein repair or turnover, membrane maintenance, and DNA repair. Genes specific to the RpoH_I_ regulon encode other proteins involved in protein maintenance and DNA repair, whereas genes specific to the RpoH_II_ regulon include proteins involved in maintaining the oxidation-reduction state of the cytoplasmic thiol pool. We used information on the members of each regulon to generate and test hypotheses about DNA sequences that determine promoter specificity of these two RpoH homologs. The observed properties of these two *R. sphaeroides* RpoH homologs illustrate how duplication of an alternative σ factor and subsequent changes in promoter recognition could have allowed convergence of transcriptional responses to separate signals. In the case of *R. sphaeroides*, we predict that these events allowed convergence of the transcriptional responses to heat shock and singlet oxygen stresses to be under control of these two RpoH paralogs.

## Results

### Defining the distinct and overlapping regulons of *R. sphaeroides* RpoH_I_ and RpoH_II_

**Figure 1:**
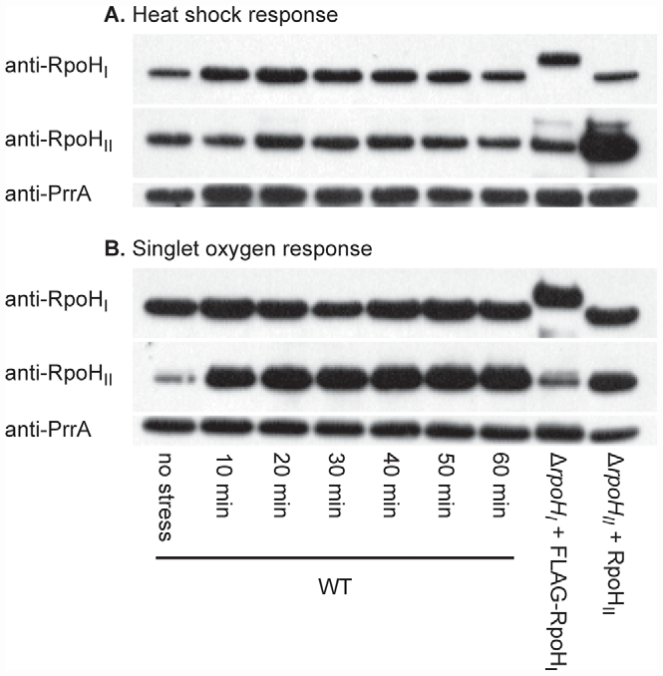
RpoH_I_ and RpoH_II_ accumulation following heat and singlet oxygen stresses. Western blots illustrating the levels of RpoH_I_ and RpoH_II_ in wild-type *R. sphaeroides* (WT) at different times following (A) a shift of temperature from 30°C to 42°C (heat shock) or (B) addition of the photosensitizer methylene blue in the presence of oxygen (singlet oxygen stress). On the same western blots, the levels of FLAG-RpoH_I_ and RpoH_II_ obtained from ectopic expression vectors used in the expression profiling and ChIP-chip experiments under normal conditions. Note that because of the addition of the FLAG polypeptide, RpoH_I_-FLAG migrates slower than the wild-type RpoH_I_. The abundance of RpoH_I_ and RpoH_II_ in wild-type cells in the absence of added stress are shown in the first lane. As a gel loading control, the membranes were also subsequently treated polyclonal antibodies against the response regulator PrrA, a control transcription factor who's expression is not known to be dependent on either of the RpoH homologs. The experiment was designed to analyze changes in levels of RpoH_I_, RpoH_II_ and PrrA before and after a stress, so the differences between panels reflect different exposure times used when developing the Western blots.

To define members of the RpoH_I_ and RpoH_II_ regulons, we monitored transcript levels and protein-DNA interactions in *R. sphaeroides* strains ectopically expressing either RpoH_I_ or RpoH_II_. To generate these strains, we constructed low copy plasmids carrying *rpoH_I_* or *rpoH_II_* under the control of an IPTG-inducible promoter [22] and conjugated them into *R. sphaeroides* mutant strains lacking *rpoH_I_* [16] or *rpoH_II_* [15], respectively. To induce target gene expression, we exposed exponentially growing aerobic cultures to IPTG for one generation before cells were either harvested to extract total RNA for analysis of transcript levels or treated with formaldehyde to prepare samples for chromatin immunoprecipitation on a chip (ChIP-chip) assays. The Western blot analysis used to measure levels of these alternative σ factors demonstrates that cells ectopically expressing RpoH_I_ and RpoH_II_ contained each protein at levels comparable to those following either heat shock or singlet oxygen stress (Figure 1). Thus, these strains can be used to characterize members of the RpoH_I_ and RpoH_II_ regulons.

As controls for this experiment, we measured the abundance of individual RpoH proteins and a control transcription factor (PrrA) [23], which is not known to be dependent on either alternative σ factor for its expression, when wild type cells were exposed to either heat or singlet oxygen stress. This analysis showed that RpoH_I_ is detectable prior to heat stress, but its levels increase 10 and 20 minutes after the shift to increased temperature (Figure 1A). RpoH_I_ levels remain elevated after the temperature shift but they decline within 60 minutes after heat shock, suggesting that as in the case of *E. coli σ^32^*, there is an initial rise in RpoH_I_ levels immediately on heat shock before they return to a new steady state level at elevated temperature [24]. RpoH_II_ was also detected prior to exposure to singlet oxygen and within 10 minutes of exposure to this reactive oxygen species, levels of this protein were increased (Figure 1B). Levels of RpoH_II_ found within 20 minutes after exposure to singlet oxygen remained relatively constant over the time course of this experiment, suggesting a continuous requirement for RpoH_II_ during this stress response (Figure 1B). The abundance of the control transcription factor PrrA did not follow these same trends, suggesting that the observed increases in individual RpoH proteins was associated with these stress responses. In addition, the abundance of individual RpoH proteins did not increase significantly to both stress responses, as expected if these increases were not due to a general increase in protein levels in response to different signals.

**Figure 2:**
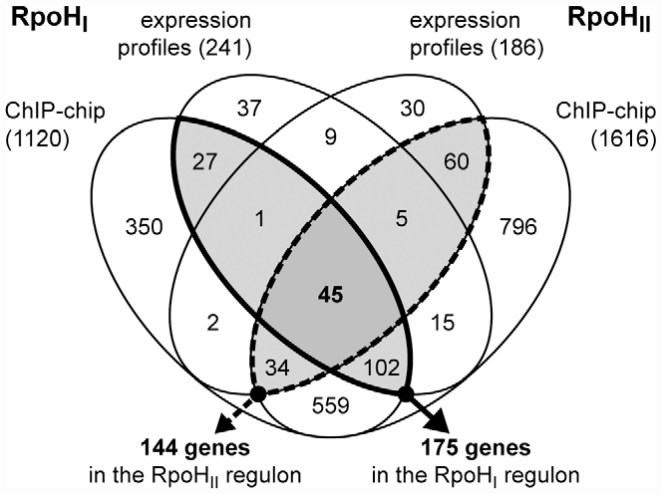
Overlap between the RpoH_I_ and RpoH_II_ regulons. Venn diagram representing the overlaps between genes that were significantly induced by the expression of RpoH_I_ or RpoH_II_, and genes whose promoters were bound by RpoH_I_ or RpoH_II_ containing RNA polymerase holoenzyme *in vivo*. The total numbers of genes identified in each study are indicated in the parentheses. The RpoH_I_ (solid outline) and RpoH_II_ (dashed outline) regulons, as defined in this study, are identified by the emphasized outlines. The total numbers of genes contained in each regulon are indicated below the arrows.

To identify transcripts that were increased in abundance as a result of RpoH_I_ or RpoH_II_ activity, we compared mRNA levels of cells expressing RpoH_I_ or RpoH_II_ ectopically to those of control cells lacking either *rpoH_I_* or *rpoH_II_*. We selected differentially expressed genes with a significance level set for a false discovery rate ≤5% and that displayed at least 1.5-fold higher transcript levels in cells expressing either RpoH family member. This analysis revealed that transcripts from 241 and 186 genes were increased by expression of RpoH_I_ and RpoH_II_, respectively (Figure 2). These two sets of differentially expressed genes have 60 genes in common.

We recognize that some of these differentially-expressed transcripts might be not be direct targets for RpoH_I_ and RpoH_II_. Therefore, to determine which of the above genes were directly transcribed by RNA polymerase holoenzyme containing either RpoH_I_ or RpoH_II_, we performed ChIP-chip assays from comparable cultures to map direct interactions of RpoH_I_ or RpoH_II_ with genomic DNA. We were able to raise specific antibodies against RpoH_II_ that performed well for the ChIP-chip assay, but repeated attempts to raise suitable antibodies against RpoH_I_ failed. Therefore, we placed a FLAG polypeptide tag [25] at the N-terminus of the RpoH_I_ protein sequence and used anti-FLAG monoclonal antibodies to perform the ChIP-chip assay. As a control we tested and showed that addition of the polypeptide tag did not alter the activity and specificity of RpoH_I_ by comparing the mRNA level profiles of cells expressing the tagged version of RpoH_I_ with cells expressing wild-type RpoH_I_ (Figure S1). In addition, other control experiments showed there was no detectable cross-reaction between FLAG-RpoH_I_ and the antibody used to precipitate RpoH_II_, and vice versa (data not shown). From the ChIP-chip analysis we identified 812 and 1353 genomic regions enriched after immunoprecipitation with antibodies against RpoH_I_ and RpoH_II_, respectively, using a significance level set for a false discovery rate ≤5%. Because the signal from a single σ factor binding site extends on average over a 1 kb region, some enriched regions may contain multiple binding sites. To increase the resolution of the putative RpoH_I_ and RpoH_II_ binding sites, we identified the modes of the ChIP-chip signal distributions within each enriched region. This adjustment increased the number of putative binding sites for RpoH_I_ and RpoH_II_ to 1085 and 1765, respectively.

We then identified all the annotated genes that contained a ChIP-chip peak within 300 base pairs upstream of their start codons as a way to define candidate genes or operons in the RpoH_I_ or RpoH_II_ regulons. Included in this list of potential regulon members were genes that are predicted to be co-transcribed using a previous computational analysis of *R. sphaeroides* operon organization (http://www.microbesonline.org/operons/) [26]. Therefore, by these criteria, the upper limits of the total numbers of genes potentially regulated by RpoH_I_ or RpoH_II_ are 1120 and 1616, respectively (Figure 2). We recognized that a significant number of the putative RpoH_I_ or RpoH_II_ promoters may not be assigned from the ChIP-chip dataset alone, especially because promoter orientation needs to be considered and that because σ factor or RNA polymerase binding events do not always promote transcription. Therefore, we refined the respective RpoH_I_ and RpoH_II_ regulons by intersecting the lists of target genes identified from the ChIP-chip analysis with the lists of candidate genes identified from the expression profiling analysis. After this intersection, we predict that the RpoH_I_ regulon contains 175 genes and the RpoH_II_ regulon contains 144 genes with 45 genes common to both regulons (Figure 2).

Upon examining the annotations of these predicted target genes, the 45 genes that are members of both the RpoH_I_ and RpoH_II_ regulons are predicted to encode mainly for functions related to the electron transport chain, protein homeostasis, and DNA repair (Table 1 and Table S1). The 130 predicted members of the RpoH_I_ regulon also encode functions in these three groups, but with a larger representation for functions associated with protein homeostasis. The 99 predicted members of the RpoH_II_ regulon include fewer proteins predicted to play a role in protein homeostasis and a larger number of proteins predicted to help maintain the oxidation-reduction state of the cytoplasmic thiol pool. However, a large number of genes in both the unique and overlapping RpoH_I_ and RpoH_II_ regulons are annotated as having no predicted functions. Overall, this analysis revealed that RpoH_I_ or RpoH_II_ activate a large set of distinct and overlapping sets of target genes.

**Table 1:** Compositions of the RpoH_I_ and RpoH_II_ regulons.

Mainrole§	Subrole†	Locus‡
**RpoHI/II regulon (45 genes)**		
Energy metabolism	Electron transport	RSP_0100, RSP_0101, RSP_0102, RSP_0103, RSP_0104, RSP_0105, RSP_0106, RSP_0107, RSP_2805
	Other	RSP_0472
Protein synthesis/fate	Degradation of proteins, peptides, and glycopeptides	RSP_0665, RSP_1076, RSP_1174, RSP_2710, RSP_2806
	Other	RSP_1825
	Protein folding and stabilization	RSP_1207
	Ribosomal proteins: synthesis and modification	RSP_0570
	Serine family	RSP_2481
Fatty acid and cell envelope	Biosynthesis	RSP_0473
	Biosynthesis and degradation of surface polysaccharides and lipopolysaccharides	RSP_2569
	Other	RSP_3601
DNA metabolism	DNA replication, recombination, and repair	RSP_2388, RSP_2966
Central intermediary metabolism	Phosphorus compounds	RSP_0013
Cellular processes	Adaptations to atypical conditions	RSP_2617
Signal transduction	Two-component systems	RSP_0847
Unknown function	Unknown function	RSP_2718, RSP_0011, RSP_0370, RSP_1025, RSP_1239, RSP_1760, RSP_2218, RSP_2421, RSP_2625, RSP_2973, RSP_3327, RSP_3599, RSP_0870, RSP_1026, RSP_1421, RSP_1840, RSP_2261, RSP_2265
**RpoHI regulon (130 genes)**		
Protein synthesis/fate	Amino acid biosynthesis	RSP_0244, RSP_0377, RSP_1475
	Degradation of proteins, peptides, and glycopeptides	RSP_0357, RSP_0554, RSP_1408, RSP_1531, RSP_1742, RSP_2412, RSP_2649
	Protein and peptide secretion and trafficking	RSP_1169, RSP_1797, RSP_1798, RSP_1799, RSP_1843, RSP_2540, RSP_2541
	Protein folding and stabilization	RSP_1016, RSP_1173, RSP_1532, RSP_1572, RSP_1805, RSP_4043
	Protein modification and repair	RSP_0559, RSP_0872, RSP_0873, RSP_0874, RSP_0923
	tRNA aminoacylation	RSP_0875
Energy metabolism	Amino acids and amines	RSP_3957
	Electron transport	RSP_0296, RSP_0610, RSP_1194, RSP_1489, RSP_1529, RSP_1576, RSP_2375, RSP_2685, RSP_2945
	Glycolysis/gluconeogenesis	RSP_0361
Fatty acid and cell envelope	Biosynthesis	RSP_0720, RSP_0929, RSP_2776
	Biosynthesis and degradation of murein sacculus and peptidoglycan	RSP_1240
	Biosynthesis and degradation of surface polysaccharides and lipopolysaccharides	RSP_0125, RSP_3187
	Degradation	RSP_0409
	Other	RSP_1889
Biosynthesis of cofactors, prosthetic groups, and carriers	Folic acid	RSP_0930
	Lipoate	RSP_2783
	Molybdopterin	RSP_0235, RSP_1071, RSP_1072
	Other	RSP_2658
	Pyridoxine	RSP_1672
Regulatory functions	DNA interactions	RSP_0014, RSP_2200
	Other	RSP_2236
	Protein interactions	RSP_4193
	Transcription factors	RSP_2410
Transport and binding proteins	Amino acids, peptides and amines	RSP_1564
	Cations and iron carrying compounds	RSP_2542, RSP_2891
	Unknown substrate	RSP_2696, RSP_2897
DNA metabolism	DNA replication, recombination, and repair	RSP_1074, RSP_2815, RSP_4199
	Purine ribonucleotide biosynthesis	RSP_2454
Cellular processes	Adaptations to atypical conditions	RSP_4198
	Detoxification	RSP_0890, RSP_1058
Central intermediary metabolism	Other	RSP_1196, RSP_1949
	Sulfur metabolism	RSP_2738
Signal transduction	Two-component systems	RSP_2130, RSP_3105
Mobile and extra-chromosomal element functions	Transposon functions	RSP_3007
Unknown function	Unknown function	RSP_0126, RSP_0362, RSP_0363, RSP_0408, RSP_0719, RSP_0999, RSP_1104, RSP_1193, RSP_1204, RSP_1238, RSP_1241, RSP_1360, RSP_1406, RSP_1549, RSP_1563, RSP_1573, RSP_1581, RSP_1615, RSP_1671, RSP_1684, RSP_1743, RSP_1852, RSP_2121, RSP_2125, RSP_2214, RSP_2219, RSP_2387, RSP_2638, RSP_2640, RSP_2641, RSP_2739, RSP_2763, RSP_2764, RSP_2816, RSP_2952, RSP_2953, RSP_3067, RSP_3068, RSP_3378, RSP_3426, RSP_3552, RSP_3597, RSP_3598, RSP_3634, RSP_3809, RSP_3810, RSP_4244, RSP_4245, RSP_4248, RSP_4305
**RpoHII regulon (99 genes)**		
Energy metabolism	Biosynthesis and degradation of polysaccharides	RSP_0482
	Electron transport	RSP_0108, RSP_0109, RSP_0110, RSP_0112, RSP_0474, RSP_2785, RSP_3212, RSP_3305, RSP_3537
	Entner-Doudoroff	RSP_2646
	Fermentation	RSP_3164
	Glycolysis/gluconeogenesis	RSP_2736, RSP_4045, RSP_4211
	Other	RSP_0392, RSP_2294
	Pentose phosphate pathway	RSP_2734, RSP_2735
	Sugars	RSP_2937, RSP_3138
Biosynthesis of cofactors, prosthetic groups, and carriers	Glutathione and analogs	RSP_3272
	Heme, porphyrin, and cobalamin	RSP_1197, RSP_1692, RSP_2831
	Menaquinone and ubiquinone	RSP_1175, RSP_1338, RSP_1492, RSP_1869
	Other	RSP_0750, RSP_0898, RSP_2314
Transport and binding proteins	Amino acids, peptides and amines	RSP_1542, RSP_3274
	Carbohydrates, organic alcohols, and acids	RSP_0149, RSP_0150
	Cations and iron carrying compounds	RSP_1546, RSP_2608
	Unknown substrate	RSP_1895, RSP_2802, RSP_3160
DNA metabolism	DNA replication, recombination, and repair	RSP_1466, RSP_2083, RSP_2414, RSP_2850, RSP_3077, RSP_3423
	Pyrimidine ribonucleotide biosynthesis	RSP_3722
Fatty acid and cell envelope	Biosynthesis and degradation of surface polysaccharides and lipopolysaccharides	RSP_1491, RSP_2163, RSP_3721
	Degradation	RSP_0119
	Other	RSP_0422, RSP_0595, RSP_0855
Regulatory functions	DNA interactions	RSP_1083, RSP_4210
	Other	RSP_0148, RSP_2631, RSP_3430, RSP_3431
	Transcription factors	RSP_0601
Cellular processes	Detoxification	RSP_1057, RSP_2389, RSP_2693, RSP_3263
	Toxin production and resistance	RSP_2803
Central intermediary metabolism	Other	RSP_0897, RSP_1258, RSP_1397, RSP_3072
	Phosphorus compounds	RSP_0782
Protein synthesis/fate	Amino acid biosynthesis	RSP_0398
	Degradation of proteins, peptides, and glycopeptides	RSP_0686, RSP_1490
	Protein folding and stabilization	RSP_1219
	tRNA and rRNA base modification	RSP_2971
Unknown function	Unknown function	RSP_0151, RSP_0152, RSP_0269, RSP_0423, RSP_0557, RSP_0799, RSP_0896, RSP_1591, RSP_1956, RSP_1985, RSP_2225, RSP_2268, RSP_3075, RSP_3076, RSP_3089, RSP_3310, RSP_3329, RSP_4144, RSP_4209

Summary of the functional annotations of members of the RpoH_I_ and RpoH_II_ regulons in *R. sphaeroides* defined by the intersections of the results from the expression profiling and chromatin immunoprecipitation experiments.

§ Classification of the functional main categories according to the JCVI-CRM database (http://cmr.jcvi.org/).

† Classification of the functional sub-categories according to the JCVI-CRM database.

‡ Unique locus identifiers for *R. sphaeroides* 2.4.1.

### Predicted differences in promoter sequences recognized by RpoH_I_ or RpoH_II_

Previous work indicated that RpoH_I_ and RpoH_II_ can recognize and initiate transcription from similar promoter sequences [14], [15], [20]. The characterization of their respective regulons also suggests that some promoters can be transcribed by both σ factors while others are specific to either RpoH_I_ or RpoH_II_. Therefore, we hypothesized that while the promoter sequences of the two σ factors may be similar, different sequence-specific interactions of RpoH_I_ or RpoH_II_ with promoter elements are the basis of promoter specificity for transcription initiation by RNA polymerase.

To overcome the limited resolution of the ChIP-chip experiment and predict determinants of promoter specificity for RpoH_I_ or RpoH_II_, we searched the regions upstream of genes in each regulon for conserved sequence elements (137 sequences for RpoH_I_ and 120 sequences for RpoH_II_). The conserved sequence elements we identified mapped to putative promoter elements that were within 100 bp of the coordinates of the modes of the distributions of the ChIP-chip signal. Thus, the predictions of these searches identified conserved sequence elements that were in agreement with the experimental data. In addition, even though we analyzed the individual RpoH_I_ and RpoH_II_ regulons independently for these motifs, the sequence alignment algorithm converged to the same sequence elements for promoters that were predicted to be recognized by both RpoH_I_ and RpoH_II_. This result is not surprising given that both σ factors have similar amino acid sequences in their DNA recognition regions and are thus expected to recognize similar promoter sequences. However, this observation supports the hypothesis that RpoH_I_ and RpoH_II_ recognize common promoter sequences in their respective target genes as opposed to distinct promoters.

**Figure 3:**
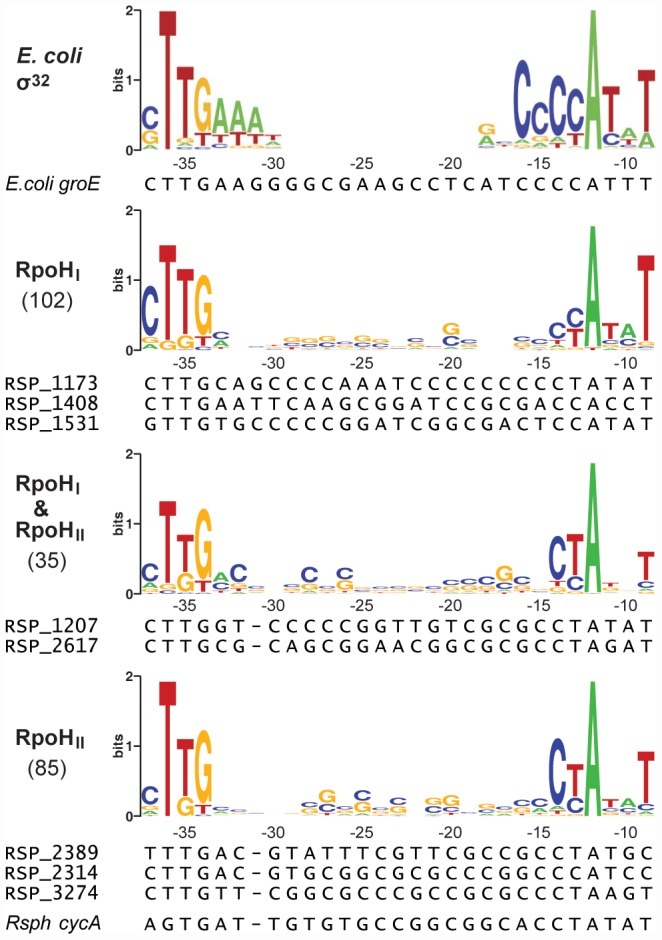
Conserved promoter sequences recognized by RpoH_I_ and RpoH_II_. The logos were constructed from promoter sequences alignments sorted into three categories according to their predicted specificity. The consensus sequence for σ^32^-dependent promoters in *E. coli*,as determined by Nomaka et al. [37], is shown as a reference. The heights of the letters represent the degree of conservation across sequences (information in bits, logos generated using WebLogo: http://weblogo.berkeley.edu/). The coordinates on the x-axes represent the positions relative to the predicted transcription start site. The numbers of promoter sequences used to create the logos are indicated in parentheses on the left of the logos. Below the logos are the sequence alignments of selected promoters that were used for direct experimental validation.

To predict specificity sequence determinants for each RpoH paralog, the putative distinct and overlapping promoter sequences were sorted into three groups according to the expression profiling and ChIP-chip data sets and converted into sequence logos (Figure 3, Table S2). The sequence logos derived from the three groups include: two groups that are preferentially or selectively bound and transcribed by either RpoH_I_ or RpoH_II_ and one group that is bound and transcribed by both σ factors. As noted above, some promoters appear to be bound by RpoH_I_ or RpoH_II_ without inducing detectable changes in transcript levels. We aligned these promoters separately to determine if they possessed unique characteristics, but no significant differences were detected (data not shown).

The conservation of a TTG motif in the −35 region in all three logos is consistent with the importance of this triplet in a previous analysis of at least one promoter known to be recognized by both RpoH_I_ and RpoH_II_ [27]. However, there was also evidence for sequence-specific elements in the logos for each RpoH paralog. In the logo for the RpoH_I_-dependent promoters, a cytosine is overrepresented at position −37 and a thymine is overrepresented at position −9. In the logo for RpoH_II_-dependent promoters, cytosine and thymine are overrepresented at positions −14 and −13, respectively.

Overall, the comparison between RpoH_I_ and RpoH_II_-specific promoter logos allowed us to identify significant differences in the promoter sequences that may be used to adjust promoter selectivity and strength for RpoH_I_ or RpoH_II_. In addition, the predicted sequence elements for RpoH_I_ or RpoH_II_ promoters are not mutually exclusive. Rather, it appears that promoter specificities for RpoH_I_ or RpoH_II_ are distributed along a gradient using a combination of specific bases at various positions of the −35 or −10 promoter elements.

### Degrees of promoter specificity of RpoH_I_ and RpoH_II_

**Figure 4:**
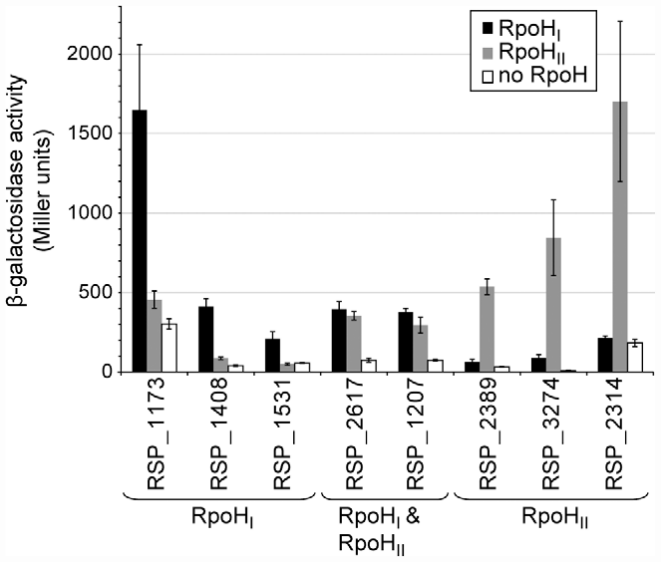
Relative activities of selected RpoH_I_- and RpoH_II_-dependent promoters. β-galactosidase activity of *lacZ* operon fusions with selected *R. sphaeroides* promoter regions monitored in tester strains expressing RpoH_I_ (black), RpoH_II_ (grey), or neither proteins. Genes are grouped according to the gene expression profiles displayed in the gene expression experiments: genes whose expressions were affected only by RpoH_I_, only by RpoH_II_, and by both RpoH_I_ and RpoH_II_. Error bars represents the standard error of the mean from three independent replicates.

To test predictions about specificity determinants derived from these logos, we cloned several putative promoters upstream of a *lacZ* reporter gene and integrated these into the genome of a *R. sphaeroides* Δ*rpoH_I_* Δ*rpoH_II_* mutant [15] via homologous recombination. The activity of each promoter was measured by assaying β-galactosidase activity in these *R. sphaeroides* reporter strains ectopically expressing either RpoH_I_ or RpoH_II_ (Figure 4) at levels comparable to those found during a stress response (see above and Figure 1). The RSP_1173, RSP_1408, and RSP_1531 promoters (which were either predicted to be members of the RpoH_I_ regulon or, in the case of RSP_1173, known to be heat inducible and transcribed by RpoH_I_ [16], had significant activity in the strain expressing RpoH_I_, but not when the same strain expressed RpoH_II_ (Figure 4). In contrast, the RSP_2314, RSP_2389, and RSP_3274 promoters (which were either predicted to be members of the RpoH_II_ regulon by our analysis or known to be induced by conditions that generate singlet oxygen [17], [18], [20]) showed activity in the presence of RpoH_II_ but not RpoH_I_ (Figure 4). Finally, the RSP_1207 and RSP_2617 promoters (which were predicted to be transcribed by both RpoH proteins and, in the case of RSP_1207, known to be transcribed by RNA polymerase holoenzyme containing either RpoH homolog [15] showed activity in cells containing either RpoH_I_ or RpoH_II_ (Figure 4). Overall, these results support predictions about members of the RpoH_I_ or RpoH_II_ regulons derived by combining the transcription profiling, ChIP-chip and computational analyses.

**Figure 5:**
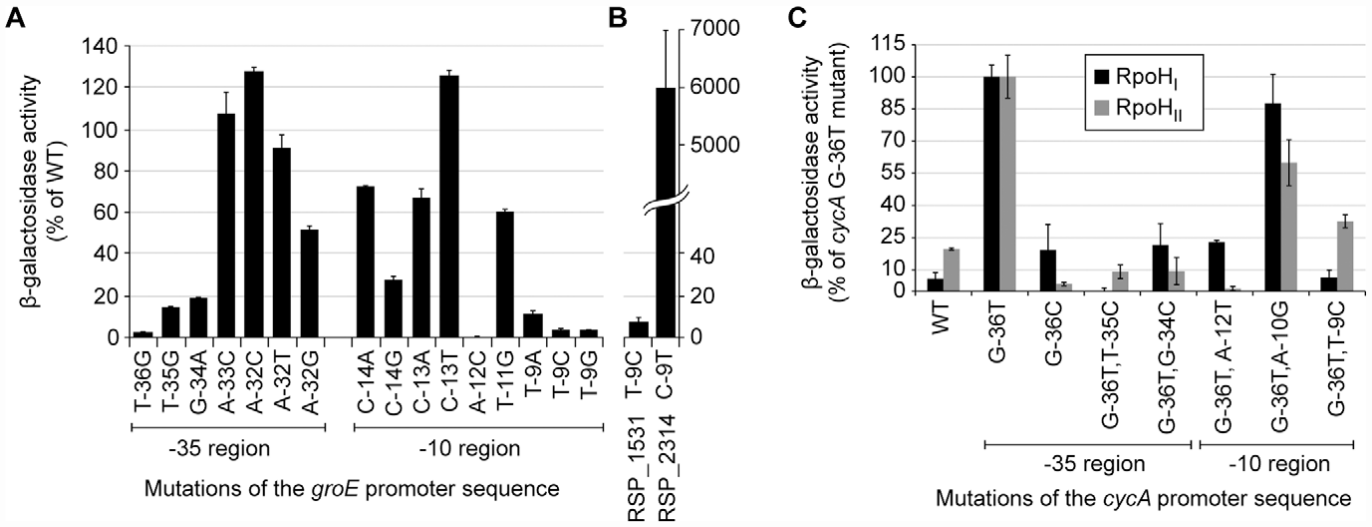
Activities of selected mutant promoters when transcribed by RpoH_I_ or RpoH_II_. β-galactosidase activity of *lacZ* operon fusions with selected mutant *E. coli* (A) or *R. sphaeroides* (B) promoters monitored in an *E. coli* tester strain expressing *R. sphaeroides* RpoH_I_. The original promoter and specific base substitutions are indicated below the x-axis. (C) β-galatosidase activity of *lacZ* operon fusions integrated into the genome containing either the wild type of indicated mutant *R. sphaeroides cycA* P1 promoter in a tester strain expressing the indicated RpoH homolog. Most of the promoter mutations were made in the G-36T *cycA* P1 background, as this promoter had activity with RpoH_I_ or RpoH_II_ than its wild type (WT) counterpart. Base substitutions are indicated on the x-axis. Error bars represents the standard error of the mean from three independent replicates.

To test the predictions about the contributions of individual bases to promoter recognition, we measured the activity of *R. sphaeroides* RpoH_I_ with an existing library of mutant *E. coli groE* promoters fused to a *lacZ* reporter in an *E. coli* tester strain [7]. The data from this analysis revealed that base substitutions in the TTG motif of the −35 region of this RpoH-dependent promoter (positions −36, −35, and −34) reduced its activity by at least 80% with RpoH_I_ (Figure 5A), as expected from the predictions of promoter logo. We also found a slight increase in promoter activity when position −32 was changed to a cytosine, even though the C-32 is not conserved in RpoH_I_ promoters. This observation is consistent with the results of a previous mutational analysis showing that *E. coli* σ^32^ prefers a cytosine at position −32 when the alanine at position 264 of its amino acid sequence is substituted to an arginine (corresponding to R267 of RpoH_I_) [28], but also suggests that the −32 position is not utilized to distinguish between RpoH_I_- and RpoH_II_-specific promoters. In the −10 region of the *groE* promoter, substitutions of the cytosine at position −14 for an adenine or guanine, the cytosine at position −13 for an adenine, or substitution of the thymine at position −11 for a cytosine, each reduced RpoH_I_-dependent promoter activity. In addition, a substitution of the adenine at position −12 for a cytosine or changing the thymine at position −9 for any other base reduced RpoH_I_-dependent activity by >90%. These observations are consistent with the conservation of a thymine at position −9 of the derived RpoH_I_ promoter logo (Figure 3).

To test the predicted requirement of RpoH_I_ for a thymine at position −9, we also analyzed the properties of two *R. sphaeroides* promoters in this *E. coli* tester strain. Activity of the RpoH_I_-dependent RSP_1531 promoter was reduced by 90% when the thymine at position −9 was changed to a cytosine, whereas the RpoH_II_-dependent RSP_2314 promoter had higher RpoH_I_-dependent activity when a thymine was placed at position −9 (Figure 5B). Therefore, this analysis confirmed that position −9 plays a critical role in promoter specificity for RpoH_I_. In conclusion, the measured effects of mutations in the *E. coli groE* promoter on RpoH_I_-dependent transcription confirmed that our models captured elements that are critical for promoter recognition by RpoH_I_.

We were unable to test activity of *R. sphaeroides* RpoH_II_ against this *groEL* promoter library in the same *E. coli* tester strain (data not shown). Instead, we generated a small set of point mutations in the P1 promoter of the *R. sphaeroides cycA* promoter (Figure 3) which was previously shown to be transcribed by both RpoH_I_ and RpoH_II_ [27] and measured activity from single-copy fusions of these mutant promoters to *lacZ* in cells that either lacked both RpoH homologs or that contained a single *rpoH* gene under control of an IPTG-inducible promoter (Materials and Methods).

By analyzing this promoter library, we found that a G to T mutation at position −36 of *cycA* P1 (G-36T) increased its transcription by both RpoH_I_ and RpoH_II_ (Figure 5C). This result is consistent with the high predicted information content for T at this position for both RpoH_I_ and H_II_ (Figure 3), as well as the previous observation that the overall increase in activity of *cycA* P1 is caused by the G-36T mutation [27]. While our RpoH_I_ and RpoH_II_ promoter models (Figure 3) predict that a C could be allowed at position −36, a G-36C mutation lowered activity with RpoH_II_ and had no positive impact on transcription by RpoH_I_ (Figure 5C). Due to the significantly increased in activity from the G-36T mutation in *cycA* P1, all of the other promoter mutations we tested were generated in this background. Mutations we tested in the −35 region, T-35C and G-34C, resulted in virtually complete loss of *cycA* P1 activity with either RpoH_I_ and RpoH_II_ when compared to their G-36T parent promoter (Figure 5C), indicating that these bases are essential for transcription initiation by both RpoH homologs. Based on the relatively low information content predicted by our models for other positions in the −35 element (Figure 3), we did not test the effects of other mutations in this region on promoter selectivity by RpoH homologs.

In the predicted −10 region, A-12 has very high information content for both RpoH_I_ and RpoH_II_, but the sequence logo suggests a T at this position might allow selective recognition by RpoH_I_ (Figure 3). Indeed, a promoter containing a T at position −12 is still active only with RpoH_I_, suggesting that A-12 is essential for RpoH_II_ activity but not RpoH_I_ activity. The T at position −9 of *cycA* P1 is also predicted to have significantly higher information content for RpoH_I_ than RpoH_II_, while a C at this position should have more information content for RpoH_II_ than RpoH_I_ (Figure 3). As predicted, we found RpoH_II_ retained significant activity after placing a T-9C mutation in the context of the G-36T *cycA* P1 promoter. Furthermore, we found that this mutation completely abolished its activity with RpoH_I_, illustrating the high information content of a T at this position for transcription by this RpoH homolog. The importance for a T at the analogous position was also observed when testing activity of mutant *E. coli groE* promoters with RpoH_I_ (T-9C mutation Figure 5A) or assaying function of the *R. sphaeroides* RSP_1531 promoter (which contains a T, Figure 5B) that is only transcribed to a detectable level by RpoH_I_ (Figure 4). Finally, we also replaced the A at position −10 of the *cycA* P1 promoter with a G, as the sequence logo suggests there to be little information content at this position for either RpoH_I_ or RpoH_II_ (Figure 3). As predicted, there is little impact of the A-10G mutation on promoter function, though activity with RpoH_II_ is more significantly reduced than that with RpoH_I_ activity (Figure 5C).

## Discussion

When organisms encounter environmental or internal stress they often increase the transcription of genes encoding proteins that help mitigate damage to cellular components. Therefore, identifying functions that are involved in transcriptional stress responses is critical to understand both the nature of the damage caused to cellular components and how organisms respond to these challenges. Singlet oxygen and increased temperature are very different phenomena, but in *R. sphaeroides* the transcriptional responses to these two stresses involve two alternative σ factors, RpoH_I_ and RpoH_II_, that each belong to the RpoH family [15], [16], [18]. Several other α-proteobacteria contain two or more members of the RpoH family that appear to control different stress responses [13], [29], [30]. However, as it is the case in *R. sphaeroides*, little is known about the target genes for these multiple RpoH homologs. In this work, we characterized genes that are directly transcribed by *R. sphaeroides* RpoH_I_ and RpoH_II_ to gain a better understanding of the biological response to heat shock and singlet oxygen stresses. We found that each of these RpoH paralogs control transcription of over 100 genes, suggesting that each of these phenomena lead to large changes in gene expression. However, we also found that there is significant overlap in the RpoH_I_ and RpoH_II_ regulons, creating an unexpectedly extensive connection between the transcriptional responses to these two signals. In addition, we investigated the characteristics of RpoH_I_- and RpoH_II_-dependent promoters. This effort allowed us to identify sequence elements that define promoter specificity for each σ factor, thereby allowing cells to selectively partition target genes for each RpoH paralog into different stress responses.

### *R. sphaeroides* RpoH_I_ and RpoH_II_ control the expression of a common set of functions

This work revealed a surprisingly extensive overlap of the RpoH_I_ and RpoH_II_ regulons even though these two homologs activate transcriptional responses to different signals in *R. sphaeroides*. This suggests that genes activated by these two pathways of the transcriptional regulation network play a role in the physiological response to both these, and even possibly, other stresses. Indeed, the genes regulated by both RpoH_I_ and RpoH_II_ encode known or annotated functions involved in protein homeostasis, DNA repair, and maintenance of cell membrane integrity (Table 1). These types of functions are central to cell viability and may be relevant for the physiological responses to multiple stresses that can have broad primary and secondary effects on cells. Indeed, the predicted functions of the overlapping members of the RpoH_I_ and RpoH_II_ regulons encode functions that are also part of the general stress response regulons for σ^S^ in *E. coli* or σ^B^ in *Bacillus subtilis* [31], [32]. Interestingly, σ^S^ homologs are mostly present in β- and γ-proteobacteria, but to date absent from sequenced genomes of α-proteobacteria like *R. sphaeroides* (http://img.jgi.doe.gov/) [33]. Thus, it is possible that the set of genes controlled by both RpoH_I_ and RpoH_II_ is part of a general stress response that is common to the heat shock, singlet oxygen and possibly other uncharacterized signals in *R. sphaeroides* [14], [15], [17], [18], [20]. This hypothesis is supported by the observation that *R. sphaeroides* and *R. elti* strains lacking both RpoH_I_ and RpoH_II_ are more sensitive to several conditions than strains lacking only one of these proteins [13], [15], [20].

In considering the scope of functions that are regulated by both RpoH_I_ and RpoH_II_, it is also important to note that this set of genes may be larger than the one we characterized because some promoters known to be transcribed by both σ factors were only marginally affected by ectopic expression of either RpoH_I_ or RpoH_II_. For example, the RSP_2310 (*groES*) promoter was shown to be transcribed by both RpoH_I_ and RpoH_II_ in previous *in vitro* experiments [14] and was detected by our ChIP-chip experiment to be bound by both RpoH_I_ and RpoH_II_, but did not meet all the criteria of our analysis. Thus, the *groES* promoter, like other promoters, may be subject to complex regulation *in vivo*.

### RpoH_I_ and RpoH_II_ each control functions specific to heat shock or singlet oxygen stresses, respectively

Our data also significantly extend the number and types of functions that are specifically controlled by RpoH_I_ or RpoH_II_ (Table 1). We expected to find specific sets of target genes because strains lacking either RpoH_I_ or RpoH_II_ displayed different phenotypes [14], [15], [17], [20]. While previous results indicated that accumulation of ∼25 proteins was dependent on RpoH_II_ [17], our data indicate that some 150 genes are directly controlled by each *R. sphaeroides* RpoH paralog.

Genes in the direct but RpoH_I_-specific regulon encode functions that are involved in protein homeostasis, maintaining membrane integrity, and DNA repair, as is found for the *E. coli* σ^32^ regulon [3] (Table 1) The RpoH_I_ specific regulon is also predicted to encode cation transporters and proteins in the thioredoxin-dependent reduction system (Table 1). Ion transporters can aid the heat shock stress response since exporting cations like iron, which may be released by thermal denaturation of damaged iron-sulfur or other metalloproteins, decreases secondary effects caused by formation of toxic reactive oxygen species [34]. The thioredoxin-dependent reduction system reduces disulfide bonds and peroxides, which are created by protein oxidation, and thereby helps maintain cytoplasmic proteins in a reduced state [35]. Inclusion of these functions in the RpoH_I_ regulon suggests that oxidative damage may be an important secondary effect of heat shock, perhaps caused by protein denaturation or permeabilization of the cell envelope. Overall, these results support the hypothesis that the function of RpoH_I_ in *R. sphaeroides* is similar to that of σ^32^ in *E. coli* for the response to heat shock stress. In addition, it is also possible that RpoH_I_ plays a role in the *R. sphaeroides* response to other forms of stress. There is precedent for roles of σ^32^ homologs in other stress responses by other bacteria since the activity of RpoH in *Caulobacter crescentus* is increased by heavy metal stress [36].

In contrast, *rpoH_II_* transcription is under direct control of a Group IV alternative σ factor (RpoE) that serves as the master regulator of the singlet oxygen stress response [18]. In addition, an *R. sphaeroides* Δ*rpoH_II_* mutant is more sensitive to singlet oxygen than a wild-type or Δ*rpoH_I_* strain [15], [17]. Therefore, members of the direct RpoH_II_-specific regulon might be expected to play an important role in the response to singlet oxygen stress. Among the genes in the RpoH_II_-specific regulon are others predicted to function in maintaining membrane integrity and performing DNA repair, both potential targets for damage by singlet oxygen. However, the RpoH_II_–specific regulon contains fewer genes encoding functions related to protein homeostasis than found in the RpoH_I_ regulon (Table 1). Other functions apparently unique to the RpoH_II_ regulon include the glutathione-dependent reduction system, which like the thioredoxin-dependent system repair oxidized protein residues and maintain a reduced cytoplasm (Table 1). Even though the thioredoxin- and gluthatione-dependent reduction systems serve similar cellular functions, they are apparently under the control of different RpoH-dependent transcriptional networks in *R. sphaeroides*. Thus, it is possible that the thioredoxin- and gluthatione-dependent reduction systems preferentially function on different oxidized substrates. Glutathione-dependent reduction systems are known to function on lipids or other types of protein oxidative damage that might be experienced by the cell following singlet oxygen damage [35]. We also found that the RpoH_II_-specific regulon includes the multi-subunit NADH:quinone oxidoreductase and genes encoding enzymes in heme and quinone biosynthesis (Table 1). Each of these functions are critical for the respiratory and photosynthetic electron transport chains of *R. sphaeroides* and are known or predicted to contain one or more oxidant-sensitive metal centers. Thus, placement of these genes in the RpoH_II_-specific regulon suggests that these membrane or bioenergetic functions are damaged by and need to be replaced in the presence of singlet oxygen. Overall, our data indicates that the RpoH_II_-specific regulon controls expression of functions in the repair of oxidized proteins and replacement or assembly of critical electron transport chain components. Furthermore, the different types of repair functions found in the RpoH_II_ regulon predict that singlet oxygen can damage numerous cellular components.

### RpoH_I_ and RpoH_II_ recognize different but compatible promoter sequence elements

Our global gene expression data, results from analysis of gene fusions, as well as previously reported *in vitro* experiments [14], [15] all indicate that RNA polymerase containing either RpoH_I_ or RpoH_II_ can recognize some promoters in common. This observation is not surprising considering that RpoH_I_ and RpoH_II_ have similar amino acid sequences in their respective promoter recognition regions and are each able to rescue growth of *E. coli* σ^32^ mutants [14]–[16]. Likewise, the sequence logos derived here revealed that the promoter sequences recognized by each of the *R. sphaeroides* RpoH homologs are similar to both each other and to that recognized by *E. coli* σ^32^ [37].

Our experiments provide definitive evidence that some promoters are transcribed either exclusively or predominantly by RpoH_I_ or by RpoH_II_. We were also able to predict and confirm the importance of bases for activity with individual RpoH homologs (particularly those in the −35 element). We have computational and experimental observations that can explain some aspects of promoter selectivity by RpoH_I_ and RpoH_II_. For example, our experiments identify T-9 and other positions in the −10 element as potential candidates in this discrimination, as one or more substitutions have larger effects on activity with individual RpoH_I_ homologs. Mutation of T-9 to any other base reduced RpoH_I_-driven expression of GroE promoter by more than 90%, and this same effect was observed using an authentic RpoH_I_ promoter from *R. sphaeroides*. Importantly, changing the −9 position of an RpoH_II_ *R. sphaeroides* promoter to T permitted expression by RpoH_I_. Together, these data suggest that T-9 is either required for or significantly enhances expression of RpoH_I_ promoters, but is likely to be less important for expression of RpoH_II_ promoters, as there is only weak conservation of -9T in RpoH_II_ promoters. Our data also predict that other bases, which are overrepresented in the RpoH_II_ promoters, could be critical for expression by that σ factor. As is the case with *E. coli* σ^32^ there are likely to be specificity determinants that lie outside the canonical −35 and −10 elements [7], [37]. Thus, additional *in vivo* and *in vitro* experiments with a larger suite of mutant promoters and a library of mutant RpoH proteins are needed to better define the determinants of promoter selectivity by RpoH_I_ and RpoH_II_.

In conclusion, our results suggest that, at least in *R. sphaeroides*, RpoH_I_ controls functions that are necessary for maintenance of protein homeostasis and membrane integrity after temperature increase and other cytoplasmic stress, similar to the well-characterized role of *E. coli* σ^32^ in the heat shock response [3]. However, we propose that, in *R. sphaeroides*, some RpoH_I_-regulated functions are also useful for survival in the presence of other forms of stress because these target genes also contain promoters that are recognized by RpoH_II_. We propose that the duplication of an ancestral RpoH protein to create a second homolog of this alterative σ factor provided *R. sphaeroides* the opportunity to connect stress response functions to another stimulus. In this model, *rpoH_II_* was placed under the control of the master regulator of the singlet oxygen stress response and the two RpoH proteins evolved to recognize somewhat different but compatible promoter elements to assure the optimal regulation of distinct but overlapping stress regulons. As a result of these events, the transcriptional responses of *R. sphaeroides* to heat shock and singlet oxygen stress were separable but allowed to converge and contain a common set of functions. It will be interesting to identify and examine other examples of such convergence across bacteria and other organisms that possess multiple homologs of RpoH or other transcription factors.

## Materials and Methods

### Bacterial strains and growth conditions

*E. coli* strains were grown in Luria-Bertani medium [38] at 30°C or 37°C. *R. sphaeroides* strains were grown at 30°C in Sistrom's succinate-based medium [39]. *E. coli* DH5α was used as a plasmid host, and *E. coli* S17-1 was used as a donor for plasmid conjugation into *R. sphaeroides*. The media were supplemented with kanamycin (25 µg/ml), ampicillin (100 mg/ml), chloramphenicol (30 mg/ml), spectinomycin (50 mg/ml), tetracycline (10 mg/ml for *E. coli* and 1 mg/ml for *R. sphaeroides*), trimethoprim (30 µg/ml), or 0.1% of L-(+)-arabinose when required. Unless noted, all reagents were used according to the manufacturer's specifications. The list of bacterial strains and plasmids used in this study are summarized in Table S3.

### Construction of plasmids for controlled expression of RpoH_I_ and RpoH_II_ in *R. sphaeroides*

Plasmids for ectopically expressing RpoH_I_ or RpoH_II_ were constructed by separately cloning the *rpoH_I_* or *rpoH_II_* genes downstream of the IPTG-inducible promoter in pIND4 [22]. DNA fragments containing *rpoH_I_* or *rpoH_II_* were amplified from *R. sphaeroides* 2.4.1 genomic DNA using oligonucleotides containing *Bsr*DI and *Bgl*II restriction sites (for RpoH_II_, RSP_0601_*Bsr*DI_F GTAGCAATGCATGGCACTGGACGGATATACCGATC, RSP_0601_*Bgl*II_R GTAAGATCTTCATAGGAGGAAGTGATGCACCTCC, and for RpoH_I_, RSP_2410_*BsrD*I_F GTAGCAATGCATGAGCACTTACACCAGCCTTC, and RSP_2410_*Bgl*II_R GTAAGATCTTCAGGCGGGGATCGTCATGCC). These resulting fragments were digested with *Bsr*DI and *Bgl*II and ligated into pIND4 that was digested with *Bse*RI and *Bgl*II to create pYSD40 (*rpoH_I_*) and pYSD41 (*rpoH_II_*), respectively. The pYSD42 plasmid expressing the FLAG-tagged version of RpoH_I_ was constructed following the same procedure but with an oligonucleotide primer containing a sequence encoding for three consecutive copies of the FLAG epitope (DYKDDDDK) at the N-terminus (RSP_2410_3FLAG_*Bsr*DI GTAGCAATGCATGGACTACAAGGACCACGACGGCGACTACAAGGACCACGACATCGACTACAAGGACGACGACGACAAGAGCACTTACACCAGCCTTCCCGCTC). pYSD40, pYSD41, and pYSD42 were conjugated into *R. sphaeroides* Δ*rpoH_I_* [16] and *R. sphaeroides* Δ*rpoH_II_* respectively.

### Western blot analysis for the expression of RpoH_I_ and RpoH_II_

To monitor levels of RpoH_I_ and RpoH_II_ after heat shock, exponential phase aerobic cultures (69% nitrogen, 30% oxygen and 1% carbon dioxide) of wild type *R. sphaeroides* strain 2.4.1 grown at 30°C, were transferred to a 42°C warm bath with samples collected before heat treatment and at 10 min time intervals after heat shock, up to 60 min. To assess induction resulting from singlet oxygen stress, similarly grown wild type cells were treated with 1 µM methylene blue and exposed to 10 W/m^2^ incandescent light with samples collected before treatment and at 10 min time intervals after treatment, up to 60 min. Exponentially growing aerobic cultures of *R. sphaeroides* Δ*rpoH_I_* and Δ*rpoH_II_* mutants carrying the pYSD40 or pYSD42 plasmids respectively, were treated with 100 µM IPTG for one generation and harvested. All cell samples were resuspended in 3 M urea containing 1× protease inhibitor cocktail (Thermo Scientific, Rockford, IL) and sonicated. Samples were centrifuged to remove debris and total protein concentration of the samples determined with a protein assay kit following the manufacturer protocol (Bio-Rad, Hercules, CA). An equal amount of total protein for each sample was loaded onto a NuPAGE acrylamide gel (Invitrogen, Carlsbad, CA) and run in 1× 4-morpholineethanesulfonic acid running buffer at 150 V for ∼90 min. Proteins were transferred to Invitrolon PVDF membranes (Invitrogen, Carlsbad, CA), which were subsequently incubated for 1 hr in 1× Tris-buffered saline, 0.1% Triton-X, and 5% milk protein. The membranes were incubated with rabbit polyclonal antibodies raised against either RpoH_I_, RpoH_II_ or PrrA. Horseradish-peroxidase-conjugated goat anti-Rabbit IgG antibody (Thermo Scientific, Rockford, IL) was used as secondary antibody for detection with Super Signal West Dura extended duration substrate (Thermo Scientific, Rockford, IL).

### Gene expression microarrays

Triplicate 500 ml cultures were grown aerobically with bubbling (30%O_2_, 69% N_2_, 1% CO_2_) until they reached early exponential phase (OD at 600 nm of 0.15). At this point IPTG (Isopropyl β-D-1-thiogalactopyranoside) was added to a final concentration of 100 µM to induce gene expression from the pIND4 derivatives. After 3 hours incubation (OD at 600 nm of 0.30), 44 ml of cell culture were collected and 6 ml of 5% v/v phenol in ethanol was immediately added. Cells were collected by centrifugation at 6,000 *g* and frozen at −80°C until sample preparation. RNA extraction, cDNA synthesis, labeling, and hybridization were performed as previously described on Genechip Custom Express microarrays (Affymetrix, Santa Clara, CA) [40]. Processing, normalization, and statistical analysis of the expression profile data were performed in the R statistical software environment (http://www.r-project.org/) [41]. Data were normalized using the affyPLM package with default settings [42]–[44]. The expression microarray data have been deposited in the NCBI's Gene Expression Omnibus [45] and are accessible through GEO Series accession number GSE39806 (http://www.ncbi.nlm.nih.gov/projects/geo/query/acc.cgi?acc=GSE39806).

### Chromatin immunoprecipitation on a chip

Cells were harvested at mid-exponential growth (OD at 600 nm of 0.30) from the same cell cultures used for the expression microarray experiment to prepare samples for a ChIP-chip assay [19]. RpoH_I_-FLAG was immunoprecipitated using commercial monoclonal antibodies against the FLAG polypeptide (DYKDDDDK ) (Sigma Aldrich, St Louis MO). RpoH_II_ was immunoprecipitated with anti-*R. sphaeroides* RpoH_II_ rabbit serum. Labeled DNA was hybridized on a custom-made tiling microarray, synthesized by NimbleGen (Roche NimbleGen Inc, Madison, WI), covering the genome of *R. sphaeroides* 2.4.1 [19]. Before data analysis, dye intensity bias and array-to-array absolute intensity variations were corrected using quantile normalization across replicates (*limma* package in the R environment) [46]. Regions of the genome enriched for occupancy by RpoH_I_ or RpoH_II_ were identified using CMARRT with a false-discovery rate ≤0.05 [47]. The ChIP-chip data have been deposited in the NCBI's Gene Expression Omnibus [45] and are accessible through GEO Series accession number GSE39806 (http://www.ncbi.nlm.nih.gov/projects/geo/query/acc.cgi?acc=GSE39806).

### Sequence analyses

DNA sequences were manipulated using custom Python scripts. Operon structure predictions for *R. sphaeroides* 2.4.1 were obtained from VIMSS MicrobesOnline (http://www.microbesonline.org/operons/) [26]. The promoter sequences predicted to be recognized by RpoH_I_ and RpoH_II_ were discovered using Bioprospector [48] set to search for bi-partite conserved sequence motifs. The promoter sequence alignments were refined using HMMER 1.8.5 [49]. The logo representations of the promoter sequence alignment were generated using WebLogo (http://weblogo.berkeley.edu/) [50], [51].

### Construction of plasmid vectors, *lacZ* reporter promoter fusions, and β-galactosidase assays to assay promoter activity in vivo

To assay the *in vivo* activity of RpoH_I_ and RpoH_II_ at target promoters, β-galactosidase assays were conducted with *R. sphaeroides* Δ*rpoH_I_* Δ*rpoH_II_* mutant strains containing individual reporter gene fusions. To construct this set of reporter strains ∼350 base pair regions upstream of putative target genes: RSP_1173, RSP_1408, RSP_1531, RSP_2314, RSP_2389, RSP_3274, RSP_1207 and RSP_2617, were amplified from genomic DNA using sequence specific primers, with NcoI and XbaI restriction sites at the ends of the upstream and downstream primers respectively. The amplified DNA fragments were purified, digested with NcoI-XbaI and then cloned in a pSUP202 suicide vector containing a promoterless *lacZ* gene (pYSD51). These Tc^r^ plasmids were then conjugated into an *R. sphaeroides* Δ*rpoH_I_* Δ*rpoH_II_* mutant [15], generating single copy promoter-*lacZ* fusions integrated in the genome. pYSD40, pYSD41 or pIND4 (empty vector) were then conjugated into each of these reporter strains. Exponential phase cultures of these strains, grown by shaking 10 mL in 125 mL conical flasks, were then treated with 100 µM IPTG for one generation and samples analyzed for β-galactosidase activity. β-galactosidase assays were performed as previously described [52]. The data, presented in Miller units, represents the average of three independent replicates.

To test bases that contribute to RpoH_I_ and RpoH_II_ promoter specificity, β-galactosidase assays were conducted in *R. sphaeroides* tester strains containing reporter gene fusions of the *cycA* (RSP_0296) P1 promoter with a variety of point mutations (see Results). These reporter strains were constructed as described above, with individual point mutations being generated by overlap extension PCR [53]. β-galactosidase assays were conducted as described above and the data represents the average of three independent replicates. Background LacZ activity from control strains for each promoter fusion containing only the empty pIND4 plasmid (i.e. not expressing either RpoH_I_ or RpoH_II_) was subtracted from the measured LacZ activity for each mutant promoter.

The construction of the *E. coli* CAG57102 mutant strain, the promoter library, and the β-galactosidase assay used to test the activity of *R. sphaeroides* RpoH_I_ *in vivo* on mutant promoters were described previously [7]. To express *R. sphaeroides* RpoH_I_ the *E. coli rpoH* gene of pSAKT32 [7] was replaced with the *R. sphaeroides rpoH_I_* gene. At least triplicate assays for β-galactosidase activity were performed on all strains.

## Supporting Information

Figure S1 Scatter plot of RpoH_I_ versus FLAG-RpoH_I_ dependent change in gene transcription levels.
(TIF)

Table S1 RpoH_I_ and RpoH_II_ target genes.
(XLS)

Table S2 Predicted promoter sequences of RpoH_I_ and RpoH_II_ target genes.
(XLS)

Table S3 Bacterial strains and plasmids.
(XLS)
