# Supplementary figures and images for: Convergence of the Transcriptional Responses to Heat Shock and Singlet Oxygen Stresses
Source: PLoS Genet. 2012 Sep 13;8(9):e1002929. doi: 10.1371/journal.pgen.1002929 (PMC3441632; doi:10.1371/journal.pgen.1002929)

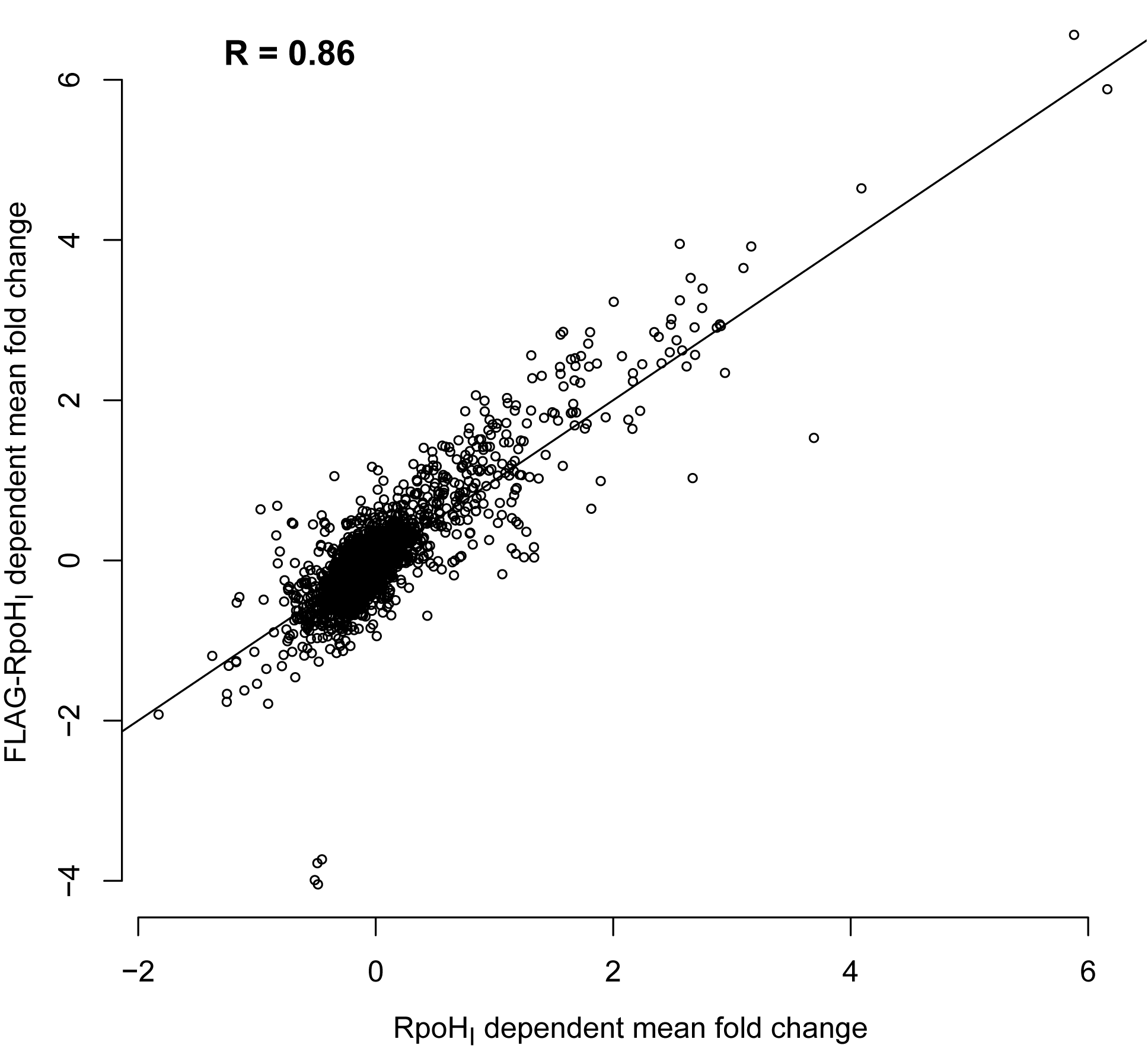

Supplement: Figure S1 — Scatter plot of RpoHI versus FLAG-RpoHI dependent change in gene transcription levels. (TIF) [file pgen.1002929.s001.tif]
